# Environmental impact assessment of tobacco farming in northern Bangladesh

**DOI:** 10.1016/j.heliyon.2023.e14505

**Published:** 2023-03-11

**Authors:** Md. Yeamin Ali, Mahir Shahrier, Abdulla - Al Kafy, Iffat Ara, Akib Javed, Md. Abdul Fattah, Zullyadini A. Rahaman, Konica Tripura

**Affiliations:** a Caritas Bangladesh, Dhaka, 1217, Bangladesh; b Department of Civil Engineering, Rajshahi University of Engineering and Technology, Rajshahi, Bangladesh; c Department of Geography & the Environment, The University of Texas at Austin, 305 E 23rd St, Austin, TX, 78712, USA; d Department of Environmental Science and Geography, Islamic University, Kushtia, 7003, Bangladesh; e State Key Laboratory of Information Engineering in Surveying, Mapping and Remote Sensing, Wuhan University, Wuhan, 430079, China; f Department of Urban and Regional Planning, Khulna University of Engineering and Technology, Khulna, Bangladesh; g Department of Geography & Environment, Faculty of Human Sciences, Sultan Idris Education University, Tanjung Malim, 35900, Malaysia

**Keywords:** Environmental impact assessment, Tobacco farming, Socioeconomic conditions, Human Health

## Abstract

Tobacco farming in Bangladesh has significant and far-reaching environmental impacts, affecting the land, water, and air. While the country has implemented tobacco control measures, the lack of monitoring and enforcement has resulted in environmental degradation and public health concerns. This study aims to document the environmental impact of tobacco farming in Bangladesh, adopting a qualitative approach to collect and analyze data. The study used focus group discussions, key informant interviews, and a structured questionnaire survey to gather data, assessing the impact of tobacco farming on the environment, socioeconomic conditions, and human health using a five-point impact assessment scale. Results illustrated that tobacco cultivation contributes to the ecosystem and natural resource degradation, leading to a loss of habitat diversity and domestic animal death. Soil erosion, water pollution, and air pollution from excessive plowing and pesticide usage have also been observed, causing skin diseases and other health issues. Despite some economic benefits, social conditions have worsened due to drug addiction and conflicts among tobacco workers. The study will help policymakers and environmentalists by highlighting the need to take action in reducing the environmental and social impacts of tobacco farming in Bangladesh. It also informs the public about the potential tobacco production and consumption risks. This study provides important insights into the adverse effects of tobacco farming in Bangladesh and emphasizes the importance of implementing appropriate measures to reduce environmental and public health impacts.

## 1 Introduction

Tobacco farming refers to the process of growing tobacco plants and manufacturing tobacco products. These plants are grown in diverse climates, ranging from tropical to subtropical regions, and are used to create a range of products, including cigarettes, cigars, and snuff [1]. Since the mid-1960s, tobacco farming has been a significant agricultural activity in Bangladesh and plays a vital role in the country's economy [2]. Although tobacco is grown on agricultural land, it is not a traditional crop that provides food or fiber. Instead, it is the primary raw material for products that are harmful to society, the environment, and human health, such as cigarettes, bidis, and other smokeless tobacco products [3]. Tobacco farming typically involves various labor-intensive activities, such as planting and harvesting tobacco leaves, curing the leaves, and packaging and transporting the finished product. Moreover, the rising demand for tobacco has led to increased deforestation in Bangladesh, and the environmental impact of tobacco farming has far-reaching and significant consequences.

Tobacco farming is a major source of income in many countries and helps to stimulate the local economy and contribute to overall economic growth. It has advantages over direct and indirect jobs, contributes significantly to the country's tax and foreign exchange revenues, and also provides food security in many rural communities [4]. But the direct link between tobacco cultivation and land causes deforestation and other environmental problems, such as large-scale greenhouse gas emissions, contributing to climate change [5]. Large-scale tobacco cultivation requires large tracts of land, leading to loss of vegetation cover, waterbodies, habitat destruction, soil pollution, and groundwater pollution [6]. Exposure to nicotine causes severe health impacts on the workers, such as headaches, dizziness, respiratory problems, and in extreme cases, death. Furthermore, it has been linked to gender discrimination, child labor, low wages, and the exploitation of indigenous populations [7,8].

Bangladesh plays a significant role in the production and consumption of tobacco in South Asia, exporting around one-third of its production [9]. Tobacco farming is a major agricultural activity in the country, and it is the second-largest agricultural sector, contributing significantly to the national economy. More than 10 million poor households in Bangladesh rely on tobacco cultivation for their livelihood, with the majority of tobacco growers being smallholder farmers who depend on the crop for their income [10]. Due to their poor socioeconomic conditions, tobacco cultivation is considered a lucrative and dependable cash crop by many farmers in Bangladesh [11]. Tobacco farming is mainly carried out in the northern and central regions of the country, with Bandarban, Dhaka, Chandpur, Rajshahi, Rangpur, Dinajpur, and Sylhet being the main tobacco-producing areas. The primary tobacco variety grown in Bangladesh is burley, used for making cigarettes, and other types, including Virginia, dark air-cured, and cigar-filler tobaccos, are also grown in the country. Tobacco cultivation requires a significant amount of water, fertilizer, and pesticides, which are often associated with environmental and health issues. The government of Bangladesh has implemented several tobacco control measures such as Smoking and Tobacco Products Usage (Control) Act - 2005, National Tobacco Control Cell (NTCC) - 2007, Tobacco Taxation – 2011, Smoke-Free Environment – 2013 and Ban on Tobacco Advertising, Promotion, and Sponsorship – 2019 to reduce the impacts of tobacco on the environment and public health [12]. However, there is still a long way to go to achieve the World Health Organization (WHO) target of a 30% reduction in tobacco use prevalence by 2025 [7].

Since the introduction of the sustainable development goals (SDGs), there has been an increasing amount of research into the impact of tobacco farming on various fields, such as social, economic, health, lifestyle, and environment. Tobacco curing alone requires 11.4 million metric tons of wood per year, and the production of rolling papers and packaging requires even more wood. According to IMC Worldwide (2012), approximately 0.08% of farmland worldwide is used for tobacco cultivation. WHO reports that tobacco use accounts for over 8 million deaths yearly. If current trends continue, it is estimated that one billion people will die due to tobacco exposure and use in the 21st century. In Bangladesh, tobacco was responsible for the deaths of almost 126,000 people in 2018, which equates to 13.5% of all deaths [7]. Recent studies have been conducted globally on various socioeconomic aspects of tobacco farming [13]. Studies have found that tobacco farming increases the risk of tobacco use and decreases adherence to tobacco control efforts [14], increases farmers' socioeconomic status while decreasing environmental sustainability [15,16], and affects the quality of life as a hazardous activity for general and hearing health [17]Other several studies have also identified the economic [18], social [19], health [20], and environmental impacts [[21], [22], [23]] of tobacco farming across the World and found similar insights.

Although all types of countries (developed, developing, and under-developed countries) experience the negative impacts associated with tobacco production, these impacts vary significantly between developed and developing countries [24]. People in developing and under-developed countries continue to farm tobacco despite challenging conditions, including low-profit margins and adverse health effects, leading to a challenging quality of life [25]. As tobacco production has shifted from developed to developing countries in recent decades [5], assessment of the environmental and social impacts of tobacco farming in developing countries is now crucial for environmental protection and justice issues. The importance of conducting this study lies in the fact that Bangladesh is a significant producer of tobacco and suffers from significant health, social, economic, and environmental impacts associated with tobacco production. However, insufficient research has been conducted on the effects of tobacco cultivation in Bangladesh that might contribute to adopting strategies that may reduce the adverse environmental, socioeconomic, and health impacts associated with tobacco farming and production.

This study investigated the impacts of tobacco farming on three sub-districts of Rangpur, Bangladesh, based on environment, health, and socioeconomic conditions. This study aims to fill this gap by providing important insights into the potential impacts of tobacco cultivation on the country's environment, health, and socioeconomic conditions. The study can help decision-makers in the country promote sustainability by reducing the adverse impacts of tobacco cultivation. This study provides valuable information on the effects of tobacco cultivation in Bangladesh that can inform global efforts to reduce tobacco use and promote sustainable development. By conducting this study, the researchers have contributed to the existing body of knowledge on the impacts of tobacco farming and provided a basis for future research in the field.

## 2 Methodology

### 2.1 Data collection

The study was conducted using primary data collection methods, including a questionnaire survey, key informant interviews (KII), focus group discussions (FGD), and case studies. Prior to conducting the questionnaire survey and FGDs, consent was obtained from all participants, and pseudo-names were used to maintain anonymity. The Department of Anthropology at the University of Rajshahi provided ethical approval for the study.

**Fig. 1:**
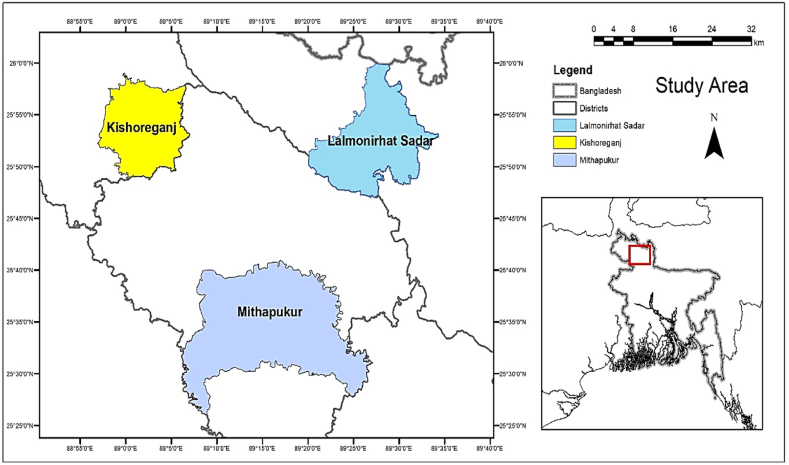
Location map of study Area.

The study utilized purposive sampling to select the study regions in northern Bangladesh. Three sub-districts in the Rangpur Division of Bangladesh (Fig. 1) were selected for the study since they are among the most significant areas where tobacco cultivation is practiced, and people are profoundly affected by it. First, three districts were chosen based on their tobacco cultivation capacity, and second, one sub-district was selected from each district, resulting in a total of three sub-districts. Nine villages from these sub-districts were selected for the study.

### 2.2 Sampling

Following the stratified purposive sampling method, three categories of people were selected as respondents. These categories include tobacco farmers and laborers, tobacco businessmen, and members of civil society (educators and politicians). Four FGDs were conducted; each group was composed of eight members based on the respondent's age, sex, and occupation. Five case studies are conducted individually to analyze the data and represent different aspects of tobacco cultivation related to their lifestyles. How many people are related to tobacco farming in the Rangpur region is still unknown. Thirty-two respondents from each category, totaling 96, were selected from the study area and among the three categories of respondents using purposive sampling for the questionnaire survey. A total of 60 KIIs were conducted to better understand the tobacco farming impact. The sampling frame is mentioned in Table 1.

**Table 1:** Sampling frame.

Category of respondents	Number of respondents
Tobacco farmer	32
Tobacco businessmen	32
Civil Society	32
KIIs	60
FGDs	32
Case Study	5
Total	193

### 2.3 Impact assessment framework

**Fig. 2:**
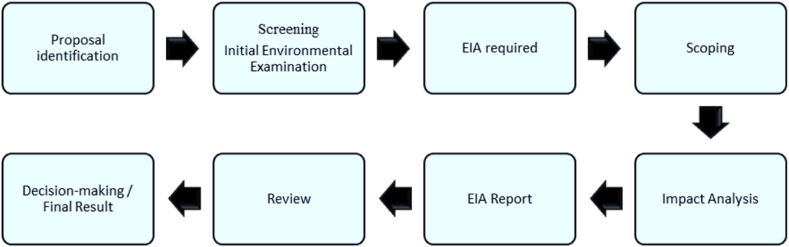
EIA process for tobacco farming.

**Fig. 3:**
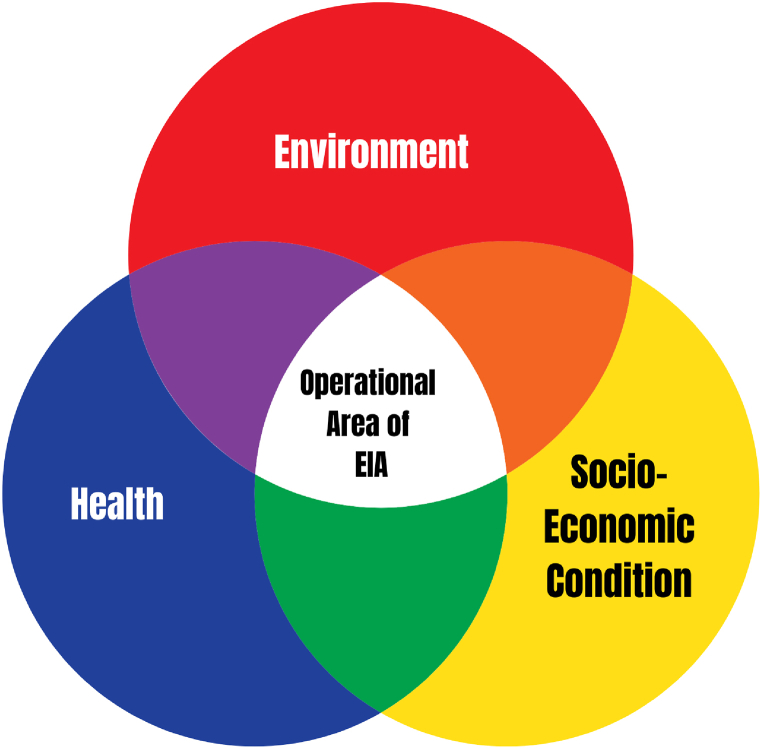
Scoping of EIA

The study identified environment, health, and socioeconomic status as parameters for assessing the environmental impact of tobacco farming (Fig. 3). Many studies have already used a similar framework with similar parameters to assess various aspects' environmental impact [26,27]. In this research, a policy-relevant approach was implied to evaluate the farming impact on the environment, which uses impacts relative to previous times. A similar approach has been used previously to assess the environmental impacts of organic farming in Europe [28]. Comparisons of this type enable us to determine whether farming performs “better” or “worse” than in the past. A qualitative assessment approach is chosen for data aggregation in appraising the impact of tobacco farming. Fig. 2 shows the Environmental Impact Assessment (EIA) workflow for tobacco cultivation.

The assessments of questionnaire surveys, FGDs, and KIIs were mainly based on people's perceptions using the Likert scale. Likert scales are well-known in social sciences and are mainly used to measure people's perceptions. We used this scale to transform qualitative data into quantitative data. A five-point assessment scale is used for the evaluation of the indicators. Table 2 shows the scale and levels, where the plus (+) sign indicates positive change, the minus (−) sign indicates negative change, and the zero (0) indicates no change in the situation over time. In detail, + + much better, + better, 0 the same, – worse, – – much worse than the previous condition. A similar model was used to understand the impact of organic farming [28]. Case studies were assessed based on respondents' opinions and observations.

**Table 2:** Assessment scale for tobacco farming impact.

Scale		Factor/Indicator
+ +	=	Much better
+	=	Better
O	=	The same
–	=	Worse
- -	=	Much worse

**Fig. 4:**
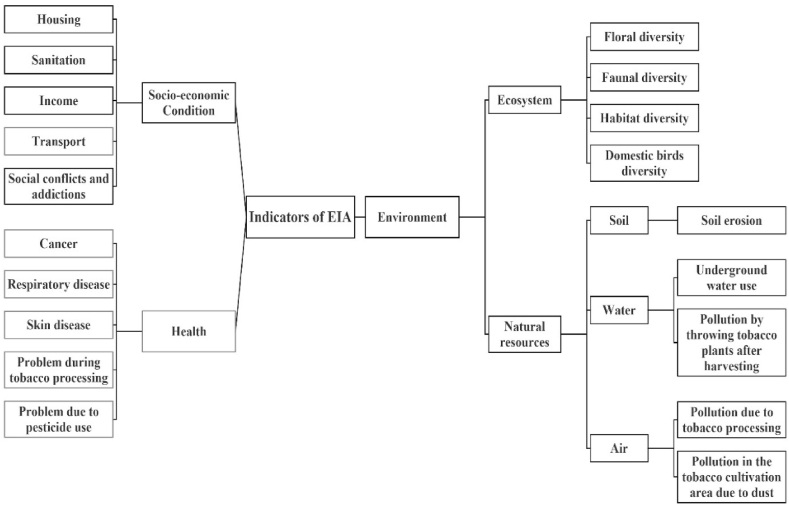
Indicators of EIA for tobacco cultivation.

The EIA measuring framework consists of three topics; environment, health, and social-economic condition. Each topic was evaluated based on indicators. These indicators were identified for tobacco farming's impact on humans and the environment (Fig. 4). A set of indicators and variables were used to assess the effect on the ecosystem, natural resources, human health, and socioeconomic conditions. Successively, verbatim quotation analysis was undertaken to extract thick and creamy data from the respondents. The term “verbatim” refers to an exact repetition without word changes [29]. To ensure the reliability of the findings, triangulation techniques were applied to data collection in order to overcome validity threats [30].

## 3 Result and discussion

### 3.1 Impact of tobacco farming

This section provides the experimental evaluation of the study comprising the review results of the indicators.

#### 3.1.1 Impact on ecosystem

The ecosystem indicator comprises floral, fauna, habitat, and domestic bird biodiversity. Table 3 shows that tobacco farming greatly worsens habitat and domestic bird diversity. Similarly, floral and faunal diversity is worse than in the previous timeframe.

**Table 3:** Assessment for tobacco farming impact on ecosystem.

Indicator	+ +	+	O	–	- -
Floral diversity				–	
Faunal diversity				–	
Habitat diversity					- -
Domestic bird's diversity					- -

Table 3 shows that every type of biodiversity has experienced a negative effect because of tobacco farming. Habitat and domestic bird diversity are more vulnerable than floral and faunal diversity. Tobacco farming is directly related to ecosystem damage in the study area. An example can be added that a study in Tanzania shows how tobacco cultivation is related to deforestation and faunal biodiversity exhaustion. Increasing demand for tobacco farming and curing caused deforestation; thus, the butterfly community is losing its variety [31]. Ecosystem disruption and soil degradation are the two main environmental effects of these practices, and as a result, there is a loss of land resources, biodiversity, and food security [32].

#### 3.1.2 Impact on natural resources

Fig. 4 shows that soil, water, and air are natural resource indicators. The study considered soil erosion in the soil category, surface and groundwater in the water category, and toxic air and dust in the air category.

Table 4 shows that natural resources such as soil, water use, and air pollution were negative, indicating that water, soil, and air quality have eroded over time. First, overall groundwater conditions deteriorate. Irrigation should be done abundantly after planting. In addition, much water is required to preserve the physical and chemical properties of the cured leaves, which puts great stress on the groundwater in the study area. Farmers usually throw tobacco plants into the water after harvest, causing water pollution in the study area. Also, fertilizers used on tobacco farms pollute surface water. Second, excessive plows increase soil erosion and airborne dust.

**Table 4:** Assessment of tobacco farming impact on natural resources.

Category	Indicator	+ +	+	O	–	- -
Water	Use of underground water in irrigation				–	
	Surface water pollution due to post-harvesting tobacco waste					- -
Soil	Erosion				–	
Air	Breathing problems during tobacco processing time due to extra CO_2_ and toxic agent				–	
	Air pollution in the tobacco cultivation area due to dust					- -

Finally, air quality is degrading due to various toxic and harmful gases from tobacco processing and leaf burning. As a result, several respiratory diseases affect residents, especially asthmatics. Long-term exposure to tobacco dust may have adverse health effects [33].

#### 3.1.3 Impact on human health

Indicators to understand the health effects were cancer (lungs and skin), skin disease, respiratory disease, health problems during processing tobacco, and health problems from pesticides. A study in Brazil discussed the health problems of tobacco farmers and various tobacco farming-related diseases [34].

Table 5 shows that human health is being affected by different diseases. Significantly, cancer and respiratory disease are worsening. Earlier studies also showed lung cancer among smokers in Bangladesh [35] and overall respiratory diseases globally [36]. Similarly, pesticides and tobacco processing caused skin diseases and other health problems, significantly worsening the situation. Excessive pesticide exposure causes adverse effects on agricultural workers’ health [37].

**Table 5:** Assessment for tobacco farming impact on human health.

Indicator	+ +	+	O	–	- -
Cancer					- -
Respiratory disease					- -
Skin disease				–	
Health problems during tobacco processing				–	
Health problems due to pesticide use				–	

The reasons for skin diseases are direct skin exposure to agrochemicals and the absence of precautionary measures while processing tobacco, like using protective gear. Besides, quite a few health problems are reported during tobacco processing and using pesticides, for instance, vomiting, weakness, headache, dizziness, proliferating perspiration and salivation, shortness of breath, episodic fluctuation of blood pressure, palpitation, an upset stomach, pain in the abdomen [38]. During the tobacco harvesting season, high levels of nicotine have been found in the urine of tobacco farm workers [39]. Overall, the study area's human health condition has deteriorated over the past few years, especially for tobacco laborers' direct contact with farming and processing. Green Tobacco Sickness (GTS) is mainly caused by nicotine exposure to the skin. Tobacco workers are recommended to wash their hands and wear protective clothing while working with tobacco [40].

#### 3.1.4 Impact on the socioeconomic condition

The most striking result of the data is the intriguing correlation between economic improvement and tobacco farming. Four indicators have been used, i.e., housing, transport, sanitation, social conflict, and addiction. Table 6 shows that income levels increased substantially among tobacco farmers. A previous study reported similar economic growth among tobacco farmers [41]. Likewise, the sanitation system is more developed than previously. Besides, housing and transportation facilities also developed, but not as much as income and sanitation. On the contrary, tobacco farming has escalated social conflict and addiction, worsening its condition.

**Table 6:** Assessment for tobacco farming impact on socioeconomic condition.

Indicator	+ +	+	O	–	- -
Income	+ +				
Housing		+			
Transport		+			
Sanitation	+ +				
Social conflict and addiction				–	

Table 6 shows that tobacco cultivation increases farm income and labor quality. The study also revealed that smoking addiction is common among youth. Addicted groups frequently create social disorders and chaos.

### 3.2 Findings of the case studies

This section describes the detailed case studies regarding the farming impact on the Rangpur region's environment, health, and socioeconomic condition.

#### 3.2.1 Case study on ecosystem

Abu Hossain (age 50) stated that plants' and animals' numbers have decreased in the last decade. Before introducing tobacco, different plant species supported varieties of bird habitats and bred in Khas (fallow) land. Nevertheless, after tobacco cultivation, animals are losing habitat, and influential people are clearing bushy species and trees from the community. Three years ago, all types of fish and other aquatic insect species in the pond had died because the day before, insecticides had been used in the tobacco field, followed by heavy rain. Therefore, the washed-away insecticide is dissolved in pond water through runoff; besides, they throw tobacco plants in the pond water after curing. Furthermore, the washed-away fertilizer from tobacco farms caused an algal bloom and killed aquatic life due to eutrophication. He also mentioned that floral diversity is disappearing and directly affecting faunal diversity because of deforestation.

The statements indicate that with the tobacco cultivation initiation, the ecosystem started weakening. Similar studies assessed the global deforestation caused by tobacco farming, which is well-established in academia [42,43]. Lecours et al. (2012) concluded that tobacco farming is responsible for deforestation, soil degradation, and negative impacts on the ecosystem [19].

#### 3.2.2 Case study on natural resource

Rahim is currently employed at a garment factory in Dhaka, Bangladesh. However, in the past, he used to cultivate tobacco on his land for over a decade. While growing tobacco, he noticed that the soil's fertility decreased, which affected the cultivation of other crops as well. Interestingly, the tobacco companies shifted from one place to another as the soil quality worsened. In contrast, Rahim's ancestors had been growing cereals and vegetables in the same land year after year without any issues. But after ten years of tobacco farming, the soil quality degraded significantly. Furthermore, deforestation caused soil erosion at a faster rate.

In addition to soil degradation, the excessive use of chemical fertilizers and insecticides also contributed to soil pollution in the study area. Rahim and several other respondents reported that pesticides contaminate both surface and groundwater. Soil absorbs these chemicals when they leach below the root zone, and heavy rainfall exacerbates this situation. Additionally, tobacco farming involves excessive land tilling, which creates dust, and when the leaves are processed, it severely affects the air quality in the surrounding areas.

This case study highlights the negative impacts of tobacco farming on natural resources such as soil, water, and air. Soil erosion, water contamination, and air pollution are detrimental to human health and the environment. Previous studies have also reported similar findings, with soil quality degradation, water pollution, and air pollution being significant issues caused by tobacco farming [44,45].

#### 3.2.3 Case studies on human health

Ronjit Roy, a 40-year-old man, lost his father to cancer and learned that tobacco was the reason behind his father's illness. He also experienced being senseless while spraying insecticide in the tobacco field and was later diagnosed with an illness caused by exposure to insecticide and tobacco. This is a common phenomenon in tobacco farming, as evidenced by a case study in Malaysia that found pesticide toxicity among one-third of tobacco farm workers [46] Meanwhile, Asma Begum, a 23-year-old tobacco farmer, fainted due to tobacco smoke and chemicals released from tobacco leaves and plants while pregnant. Her family members also experienced respiratory problems and eye infections caused by prolonged exposure to dust particles from roadside tobacco farming.

These cases demonstrate that tobacco farming significantly impacts human health, not just through smoking or tobacco intake but also through exposure to pesticide use and processing. The literature review recommends hand washing and using protective clothes to mitigate the effects of nicotine exposure [40].

#### 3.2.4 Case study on the socioeconomic condition

According to Forman Ali, aged 47, road infrastructure in their childhood was unpaved, but now there have been remarkable developments in road infrastructure, and new roads have been constructed to transport tobacco-related products. The tobacco industry's enormous benefits, ready cash, flourishing market, and growing demand have enabled them to influence the renovation of transport networks. Tobacco farmers also earn hard cash quickly, which allows them to improve their housing conditions and sanitation facilities. However, drug addiction is rapidly increasing in the study area. The availability of cheap tobacco products has led to an increase in smoking habits. Besides, the youth have easy access to various drugs, which is leading to a moral decay among them. Similar studies have shown that primarily economic benefits, such as tobacco companies' incentives to farmers, a guaranteed market, and profitability, play a crucial role in tobacco farming [44,47],.

It is evident that tobacco farming has brought economic prosperity in several aspects, which has led to changes in people's lifestyles, including an upgrade in living standards and promising infrastructural development. However, moral decay is undesirable and manifests in the youth's drug addiction. Gradually, the young generation is becoming disrespectful toward their elders [48].

## 4 Conclusion

The impact of tobacco cultivation around the World is far-reaching and devastating. This study provides evidence of the significant adverse effects of tobacco farming on the environment, health, and social conditions of the three sub-districts of Rangpur, Bangladesh. The findings highlight that tobacco farming is responsible for deforestation, soil degradation, water pollution, and other environmental damages that adversely affect ecosystems and natural resources. Additionally, tobacco farming has resulted in serious health problems, including respiratory and skin diseases, due to agrochemical pollution and tobacco use. The study also reveals the adverse social impacts of tobacco farming, such as social conflict and drug addiction.

Despite the economic benefits of tobacco farming, the study concludes that the adverse impacts of tobacco cultivation far outweigh its benefits. The study reveals that many farmers prefer tobacco farming despite being aware of the negative effects, emphasizing the need for alternative livelihood options and sustainable farming practices. Furthermore, the study contributes to the scientific literature by filling the gaps in understanding the adverse effects of tobacco cultivation in Bangladesh and the relationship between the environment and tobacco.

The findings of this study have important implications for policymakers, government, NGOs, and related organizations to address the identified difficulties and promote advocacy efforts to reduce the negative impacts of tobacco cultivation. By highlighting the far-reaching and devastating impacts of tobacco farming, this study emphasizes the need for a comprehensive approach involving collaboration between various sectors to promote sustainable development and ensure a healthier and more equitable future for all.

## Author contribution statement

All authors listed have significantly contributed to the investigation, development and writing of this article.

## Funding statement

This research did not receive any specific grant from funding agencies in the public, commercial, or not-for-profit sectors.

## Data availability statement

Data will be made available on request.

## Declaration of interest's statement

The authors declare no conflict of interest.

## Ethical approval

The ethical approval for conducting the study was taken from the Department of anthropology, University of Rajshahi.

## Consent to participate

Taken from all the participants.

## Consent to publish

Taken from all the participants. We used pseudo names in the text.
