# Corrigendum to “Environmental impact assessment of tobacco farming in northern Bangladesh” [Heliyon Volume 9, Issue 3, March 2023, Article e14505]

**DOI:** 10.1016/j.heliyon.2025.e43358

**Published:** 2025-05-20

**Authors:** Md Yeamin Ali, Mahir Shahrier, Abdulla Al Kafy, Iffat Ara, Akib Javed, Md Abdul Fattah, Zullyadini A. Rahaman, Konica Tripura

**Affiliations:** a Caritas Bangladesh, Dhaka, 1217, Bangladesh; b Department of Civil Engineering, Rajshahi University of Engineering and Technology, Rajshahi, Bangladesh; c Department of Urban and Regional Planning, Rajshahi University of Engineering and Technology (RUET), Rajshahi, 6204, Bangladesh; d Department of Environmental Science and Geography, Islamic University, Kushtia, 7003, Bangladesh; e State Key Laboratory of Information Engineering in Surveying, Mapping and Remote Sensing, Wuhan University, Wuhan, 430079, China; f Department of Urban and Regional Planning, Khulna University of Engineering and Technology, Khulna, Bangladesh; g Department of Geography & Environment, Faculty of Human Sciences, Sultan Idris Education University, Tanjung Malim, 35900, Malaysia

In this article, the authors omitted the reference number and date that approval was obtained from the statement in their article regarding ethical approval for the research presented in this publication, which is required under the policies of the journal. The authors confirm that ethical approval was obtained from The Department of Anthropology at the University of Rajshahi for the research under the following reference number: IRBRUDA20210901 dated September 20, 2021, and clarified the way that consent was obtained was written informed consent. Furthermore, reference 45 is found to be a duplication of reference 23. Reference 38 is found to be a duplication of reference 20.

The original participant consent and ethical statement from section ‘2.1. Data collection’ is below:

Prior to conducting the questionnaire survey and FGDs, consent was obtained from all participants, and pseudo-names were used to maintain anonymity. The Department of Anthropology at the University of Rajshahi provided ethical approval for the study.

The original references 20, 23, 38 and 45 are below:

[20] M.Y. Ali et al., Comparative occupational health risk between tobacco and paddy farming people in Bangladesh, SSM-Mental Health 2 (2022), 100061.

[23] M.Y. Ali, M.F. Islam, M.R. Rahman, M.K. Sheema, M.R. Akhtar, Tobacco farming in Bangladesh and its impact on environment, IOSR J. Environ. Sci. Toxicol. Food Technol. 9 (12) (2015) 27–33.

[38] M.Y. Ali et al., Comparative occupational health risk between tobacco and paddy farming people in Bangladesh, SSM-Mental Health 2 (2022), 100061.

[45] M.Y. Ali, M.F. Islam, M.R. Rahman, M.K. Sheema, M.R. Akhtar, Tobacco farming in Bangladesh and its impact on environment, IOSR J. Environ. Sci. Toxicol. Food Technol. 9 (12) (2015) 27–33.

The ethical statement has been updated to include the approval reference number and date of approval as well as clarifying that written informed consent was obtained. References 38 and 45 should be excluded from the reference list. Citation 38 in section ‘3.1.3. Impact on human health’ should refer to reference 20 and citation 45 in section ‘3.2.2. Case study on natural resource’ should refer to citation 23.

The correct version of the ethical statement in section ‘2.1. Data collection’ should be as below:

Prior to conducting the questionnaire survey and FGDs, **written informed** consent was obtained from all participants, and pseudo-names were used to maintain anonymity. The Department of Anthropology at the University of Rajshahi provided ethical approval for the study **with the approval number: IRBRUDA20210901**, **dated September 20, 2021.**

The ***authors*** apologize for the errors.

## Declaration of competing interest

The authors declare no conflict of interest.

